# The Overnight Effect of Dietary Energy Balance on Postprandial Plasma Free Amino Acid (PFAA) Profiles in Japanese Adult Men

**DOI:** 10.1371/journal.pone.0062929

**Published:** 2013-05-07

**Authors:** Manabu Nishioka, Akira Imaizumi, Toshihiko Ando, Osamu Tochikubo

**Affiliations:** 1 Liberty Square Clinic, Comfort Medical Foundation, Kawasaki, Kanagawa, Japan; 2 Institute for Innovation, Ajinomoto, CO., Inc., Kawasaki, Japan; 3 Department of “AminoIndex”, Ajinomoto, CO., Inc., Tokyo, Japan; 4 Kanagawa Health Service Association, Yokohama, Japan; Gentofte University Hospital, Denmark

## Abstract

The plasma free amino acid (PFAA) profile is affected by various nutritional conditions, such as the dietary energy balance. Regarding the clinical use of PFAA profiling, it is of concern that differences in food ingestion patterns may generate systematic errors in a plasma amino acid profile and constitute a confounding factor in assessment. In this study, the overnight impact of the dietary energy balance on the postprandial plasma amino acid profile was investigated to elucidate in particular the effects of high protein meals typical in Japanese cuisine. We conducted diet-controlled, crossover trials in eleven healthy male volunteers aged 40–61 y. They consumed either a normal meal (meal N) or high protein meal (meal H) at dinner. Forearm venous blood was collected, and plasma amino acid concentrations were measured before dinner and the next morning. We found that a high protein meal in the evening that contained 40% energy would significantly increase the PFAA concentration the next morning, even more than 12 hours after the meal. Among amino acids, the most significant difference was observed in the branched-chain amino acids (BCAAs) and in some urea-cycle related compounds. If the subject consumed the high protein diet at dinner, the PFAA profile after overnight fasting might be still affected by the meal even 12 hours after the meal, suggesting that the PFAA profile does not reflect the subject's health condition, but rather the acute effect of high protein ingestion.

## Introduction

Levels of plasma free amino acid (PFAA) concentrations are determined by various factors. It is well known that PFAA balance is altered in subjects with various diseases and malnutrition, such as inborn metabolic disorder [1], liver fibrosis [2], diabetes [3], obesity [4], renal failure [5], cancer [6], [7], [8], [9], inflammatory bowel disease [10], cachexia or malnutrition caused by serious diseases or aging [11], [12], [13], [14], [15] in humans and in laboratory rodents. These reports demonstrate that the multivariate analysis of plasma free amino-acid profiles is a promising and versatile method for diagnosing various diseases [2], [6], [7], [16], [17].

In addition, technologies have recently been developed to analyze amino acids with high accuracy enough to use clinically. For example, new method to measure PFAA profiles using high-performance liquid chromatography (HPLC)–electrospray ionization (ESI)–mass spectrometry (MS) has been established [18], [19], [20]. For example, several studies demonstrated that PFAA profiling is effective for evaluate the risks of metabolic disorder such as diabetes, cardiovascular diseases, or visceral fat obesity [4], [21], [22], [23]. Moreover, PFAA profile-based cancer screening service, named “AminoIndex® Cancer Screening” service in Japan [24]. These studies demonstrate that PFAA profiling using MS technology as “focused metabolomics” has become powerful tool both for monitoring human health condition and prediction of future health risk.

However, plasma amino acid profiles also exhibit diurnal fluctuations [25], [26], [27] and a circadian rhythm [28], [29]; they are largely dependent on the food ingested [25], [26], [27] and various hormones such as insulin [26], [30], even in healthy subjects. Therefore, if the plasma amino acid profile were used for the diagnosis of various diseases, it is of possible concern that differences in food ingestion patterns could generate systematic errors in the plasma amino acid concentration and constitute a confounding factor. Indeed, several studies have reported that the dietary energy balance, in particular a high protein diet, has not only acute but also cumulative effects on plasma amino acid profiles in humans [25], [26], [27], [30], [31], [32].

To avoid these errors, it is ideally necessary to standardize the life mode, including the food ingestion, of all subjects. However, even if this were possible in well-controlled laboratory experiments using rodents, it would be impossible in clinical studies with humans, as their life modes and dietary patterns vary. Therefore, it is necessary to examine the effects of the dietary energy balance on postprandial plasma amino acid profiles under practical conditions.

Although several studies have demonstrated that postprandial amino acid profiles were affected by dietary energy balance, especially by the protein:fat:carbohydrate (PFC) balance and by the amount and species of protein ingested [25], [26], [27], [30], [31], [32], there has been no data assessing their impact after overnight fasting. In general, plasma amino acid is measured after overnight fasting because the influence of diet is considered to have declined at that time under typical dietary patterns. However, if a subject has an extraordinarily biasing dinner, the PFAA profile might be somewhat unnatural, even after overnight fasting. In this study, the overnight impact of the dietary energy balance on postprandial PFAA profiles was investigated to elucidate in particular the effects of the high protein meals that are typical in Japanese cuisine.

## Subjects and Methods

### Subjects and Ethics

Eleven normal, healthy male volunteers without apparent diseases who were undergoing periodic health checkup at the Kanagawa Health Service Association were recruited and gave their written informed consent for inclusion before they participated in the study. All data were analyzed anonymously throughout the study. The study was conducted in accordance with the Declaration of Helsinki, and the protocol was approved by the ethics committees of the Yokohama City University. Their ages ranged from 40 to 61 years old (mean 48.3±7.4), and their body mass indices (BMI) ranged between 19.6 and 29.3 (mean 23.1±2.7). These characteristics are summarized in Table 1.

**Table 1:** Characteristics of the subjects included in this study.

Subject	Age,y	Height,cm	Weight, kg	BMI
**A**	51	172	58	19.6
**B**	40	174	74	24.4
**C**	61	173	73	24.5
**D**	48	174	69	22.8
**E**	46	162	56	21.3
**F**	41	183	98	29.3
**G**	50	168	69	24.4
**H**	59	164	64	23.8
**I**	40	163	59	22.2
**J**	42	176	68	22.0
**K**	53	182	65	19.6
**Mean**	48.3	171.9	68.5	23.1
**SD**	7.4	7.1	11.4	2.7

### Test Meals

Test meals were planned according to typical Japanese cuisine. Meals were individually prepared and served to each subject. The normal meal (meal N) provides 3770 kJ of energy, with 15% of the energy provided by protein, 25% as fat, and 60% as carbohydrates. Chicken, tuna, shrimp, and soybeans provided the protein. The remainder was provided by vegetables, steamed rice, and fruits. The high protein meal (meal H) provides 4850 kJ of energy, with 40% of the energy given as protein, 30% as fat, and 30% as carbohydrates. The components of the high protein meals were almost the same as those of normal meals. The most significant increase in protein content was attributed to chicken, which was present at 80 g in the normal meal and at 375 g in the high protein meal. The nutrient compositions and energies of each meal were summarized in Table 2.

**Table 2:** Summary of the energy balance and the amount of energy of the provided meal.

Meal	Energy intake, KJ	Protein, g	Fat, g	Carbohydrate, g	P:F:C, % of energy intake
**N**	3767	34	25	129	15∶25:60
**H**	4851	116	39	77	40∶30:30

### Study Design

This study was designed to assess the effect of the energy balance of the dinner on the PFAA profile the next morning. All volunteers were subjected to a crossover study performed after a three-week interval. Subjects ate meal N or meal H until 10∶00 pm on the first day. Then, they slept at the hotel and remained there until 5∶30 pm on the second day. At that point, they moved to the clinic near the hotel where they remained until 10∶00 am on the second day. Blood samples (5 ml) were collected at 8∶30 pm on the first day (pre), 6∶00 am (8 hr after the meal), 8∶00 am (10 hr after the meal), and 10∶00 am (12 hr after meal).

Eating and drinking was restricted, with only drinking water and Japanese tea served during the study. Subjects were asked to rest quietly in intervals between blood sampling.

### Sample Collection and Preparation

Blood samples (5 ml) were collected from forearm veins into tubes containing EDTA-2Na (Termo, Japan). After collection, blood samples were cooled immediately with ice, and plasma was separated at 3,000 rpm ×15 minutes at 4°C.The plasma was deproteinized in a final concentration of 3% sulphosalicylic acid for 30–120 minutes. All samples were stored at −70°C until measurement.

### Amino Acid Analysis

Amino acid concentrations were measured by an automatic amino acid analyzer (L-8800; Hitachi, Tokyo, Japan). Amino acids were separated by cation-exchange chromatography and detected spectrophotometrically after a postcolumn reaction with a ninhydrin reagent. The measurement procedure was performed at SRL Inc., Tokyo, Japan. The following basic amino acid and related molecules (twenty-four compounds) were measured and used in the analysis: alanine(Ala), alpha-aminobutyric acid(ABA), arginine(Arg), asparagine(Asn), citrulline(Cit), cystine(Cys), glutamic acid(Glu), glutamine(Gln), glycine(Gly), histidine(His), isoleucine(Ile), leucine(Leu), lysine(Lys), methionine(Met), ornithine(Orn), phenylalanine(Phe), proline(Pro), serine(Ser), taurine(Tau), threonine(Thr), tryptophan(Trp), tyrosine(Tyr), and valine(Val). The total AA was calculated as a summation of these plasma levels. The plasma levels of the amino acids were expressed in µM.

### Calculation of PFAA Profile Based Indices

PFAA profiles were substituted into Fischer ratio defined as (Tyr+Phe)/(Val+Ile+Leu), Tyr/LNAA defined as Tyr/(Val+Ile+Leu+Tyr+Phe), and Trp/LNAA defined as Trp/(Val+Ile+Leu+Tyr+Phe), respectively. PFAA profile was also substituted into the lung cancer discriminating function previously determined [6].

### Statistical Analysis

#### Mean and SD

PFAA concentrations were given as the mean ± SD.

#### One-way analysis of variance (1-way ANOVA)

A 1-way ANOVA was performed to assess the effect of time on the postprandial PFAA concentrations of each meal.

#### Two-way analysis of variance (2-way ANOVA)

A 2-way ANOVA and a Bonferroni *post hoc* test were performed to assess the effect of the meal type on pre- and postprandial PFAA concentrations.

#### Paired *t*-test

A paired *t*-test of preprandial PFAA concentrations between the first- and second- crossovers was performed to confirm that the preprandial PFAA profiles were stable.

For all statistical analyses, significance was set at *p*<0.05.

### Software

All of the analyses were performed using MATLAB (The Mathworks, Natick, MA), and GraphPad Prism (GraphPad Software, La Jolla, CA).

## Results

### Preprandial PFAA Concentrations

Table 1 represents the characteristics of the subjects in this study. There were no obvious diseases in any of the participants.

As long as there were no significant changes in lifestyle or health status, we anticipated that PFAA profiles would be quite stable. To confirm this, preprandial PFAA profiles were compared between the first crossover and the second crossover, which occurred after a three-week interval. No significant difference in preprandial PFAA concentration was detected with a paired *t*-test except for Cys (Table 3). No significant difference in the preprandial PFAA before the ingestion of a meal was detected between meal N and meal H with a paired *t*-test (Table 4). Therefore, it was strongly suggested that the preprandial PFAA profiles for subjects at the same time were stable.

**Table 3:** Preprandial PFAA profiles of 1^st^ and 2^nd^ crossover.

Aminoacid	1st crossover		2nd crossover	
	Mean	SD	Mean	SD
**Tau**	47.7	5.2	48.1	4.2
**Thr**	112.5	14.7	119.8	23.3
**Ser**	109.8	17.5	107.8	15.4
**Asn**	43.3	4.0	46.3	5.7
**Glu**	47.6	14.5	51.1	14.9
**Gln**	573.6	54.0	550.0	42.6
**Pro**	146.3	28.9	141.4	25.8
**Gly**	201.7	28.8	204.1	23.9
**Ala**	319.9	51.7	321.5	54.6
**Cit**	35.2	6.1	34.5	6.8
**ABA**	25.9	6.4	26.5	4.6
**Val**	230.1	23.5	228.8	35.7
**Cys**	42.3	6.6	47.3	7.2*a*
**Met**	25.3	1.8	27.7	5.9
**Ile**	71.7	7.3	80.2	16.5
**Leu**	129.1	8.1	138.9	23.4
**Tyr**	59.5	8.4	58.5	8.9
**Phe**	64.8	7.7	69.5	8.5
**His**	78.4	6.1	78.0	8.9
**Trp**	49.5	5.3	51.1	10.6
**Orn**	50.2	9.4	52.0	9.4
**Lys**	177.7	21.0	179.1	61.9
**Arg**	95.6	11.2	94.1	14.8

*a* : Significant at p<0.05 with paired *t*-test.

**Table 4:** PFAA profiles of meal N-ingested subjects and meal H-ingested subjects.

Amino acid	Meal	Pre		6∶00		8∶00		10∶00	
		Mean	SD	Mean	SD	Mean	SD	Mean	SD
**Tau**	N	47.1	3.9	59.5	8	51	6.6	46.8	5.1
	H	48.6	5.3	60.2	5.7	50	5.4	50	4.6
**Thr**	N	119.2	17.7	135.7	17.4	135.9	17.4	133.5	19.4
	H	113.1	21.3	217.8	40.2*c*	174.2	34.1*b*	163.3	32.3*a*
**Ser**	N	109.6	17.6	111.5	16.1	113.3	18	113	17.1
	H	108	15.3	129.7	22.3	104.7	14.4	106.1	13.5
**Asn**	N	45.3	5.7	49.5	6.1	49.1	6.7	49.7	7.6
	H	44.4	4.6	62.6	8.9 *c*	46.4	5.9	45.8	5.5
**Glu**	N	48.3	12.4	51.2	18.9	41.1	14.1	41.9	14.6
	H	50.4	16.8	68.8	13.7*a*	45	12	43.6	10.2
**Gln**	N	567.4	44.1	571.8	55.4	583.2	62.6	596.3	56.9
	H	556.1	55	588.9	61.7	551	48.2	568.2	49.1
**Pro**	N	150.5	26.5	142.5	25.6	140.3	25.7	135.6	26.9
	H	137.3	26.7	199	36.9*c*	160.6	27.6	150.2	30.7
**Gly**	N	203.9	28.5	216.5	32	216.5	31.6	217.4	32.2
	H	202	24.3	215.1	34.7	184.6	27.4	190.2	27.6
**Ala**	N	324.7	50.7	353.6	73.8	361.7	84.1	359.8	90.2
	H	316.8	55.2	403.5	61.6	327.9	55.2	320.9	65.7
**Cit**	N	36.7	6.5	37.4	6.1	32.9	4.7	30.7	3.2
	H	33	5.8	58.7	13.6*c*	41.6	9.6*a*	35.2	6.8
**ABA**	N	26.5	6.7	27.2	5.1	28.2	4.3	30.3	6.1
	H	25.9	4.1	33.7	5.3*a*	34.4	5.9*a*	36	6.1
**Val**	N	239.5	31.6	252.3	19.1	240.5	19.9	234.2	16.2
	H	219.3	24.6	469.9	52*c*	367.3	34.7*c*	327.5	31.3*c*
**Cys**	N	45	4.8	44.3	6.1	45.1	5.4	43.8	5.5
	H	44.6	9.2	44.2	8.5	40.1	8.5	39.7	8
**Met**	N	26.7	4.8	30.4	3.4	29.6	3.8	30.1	4.5
	H	26.4	4.3	66.4	13.5*c*	40.2	7.6*b*	32.9	5.7
**Ile**	N	80.4	15	85.8	7	75.9	8.1	71.4	7.5
	H	71.5	10	200.9	36.5*c*	119.2	19.3*c*	93.7	13.6*a*
**Leu**	N	136	20.1	144	8.8	134	11.7	135.2	14.3
	H	132	15.8	314.9	51*c*	195.2	26.9*c*	164.3	19.5*a*
**Tyr**	N	59.4	8.1	66.6	8.2	62.4	8.4	60.6	10.9
	H	58.6	9.3	109.3	15.3*c*	74.5	9*a*	63.6	10.9
**Phe**	N	67.8	8.6	67.3	5.8	67	8.8	66.6	8.4
	H	66.6	8.3	95	17*c*	69.8	9.3	65.6	8
**His**	N	78.9	7.9	83.8	8.2	82.2	6.5	83.9	7
	H	77.5	7.3	98.6	14.7*b*	77	9.2	78.9	8.9
**Trp**	N	50.3	10	58.7	10.5	59.4	10.2	59.7	11.3
	H	50.3	6.4	82.2	7.6*c*	59.9	7.1	57.2	9.1
**Orn**	N	53.7	9.9	56.2	9.1	50.3	7.7	47.9	6.9
	H	48.6	8.1	116.2	25.9*c*	83.6	17.7*c*	67.7	12.7*b*
**Lys**	N	169.7	60.4	210.7	27	211.9	23.7	222.2	23.8
	H	187	21.4	350.6	52.4*c*	237.4	30	224.6	20.3
**Arg**	N	96.7	12.8	101.1	12	99.6	12.5	102.3	11.4
	H	93	13.2	163.7	28.9*c*	110.5	15.2	102.6	14.2

Each value indicates the plasma concentration expressed as µM.

Significant at *p*<0.05 (*a*), *p*<0.01 (*b*), and *p*<0.001(*c*) with paired *t*-test.

### Effect of a High Protein Diet on the Postprandial PFAA Profile

**Figure 1:**
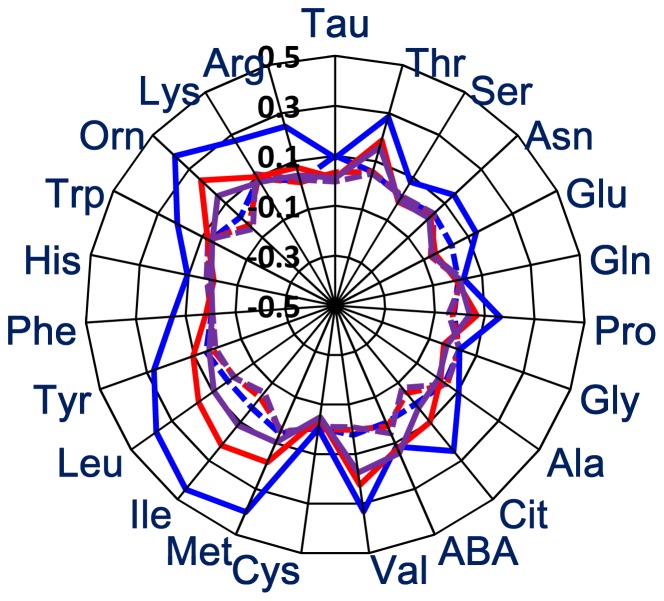
Postprandial PFAA balance in meal N ingested subjects (dotted lines) and meal H-ingested subject (solid lines) at 6∶00 (blue), 8∶00 (red), and 10∶00 (purple) a.m. Axis shows the log10 of the ratio of the plasma concentration of each amino acid at each sampling time per preprandial plasma concentration.

The effects of high protein diet ingestion on PFAA profiles were evaluated. The concentrations of most amino acids rose significantly higher in the meal H-ingested group than in the meal N-ingested group (Fig. 1, Table 4). In the meal H-ingested group, the postprandial PFAA balance was also altered substantially, compared to the preprandial PFAA balance, while the PFAA balance was stable between pre- and postprandial conditions in the meal N-ingested group (Fig. 1).

Significantly higher plasma levels of the following amino acids were observed in meal H than in meal N at the following times: 6∶00 a.m.: Thr, Asn, Glu, Pro, Cit, ABA, Val, Met, Ile, Leu, Tyr, Phe, His, Trp, Orn, Lys, and Arg; 8∶00 a.m.: Thr, ABA, Val, Met, Ile, Leu, Tyr, Orn, and Lys; and 10∶00 am: Thr, Pro, Cit, Val, Ile, Leu, and Orn (Table 4). Among them, in the meal H-ingested group, branched chain amino acids (BCAAs; Val, Ile, and Leu), urea cycle-related amino acids (Orn), and Thr remained at significantly higher levels, even at 10∶00 am (more than 12 hours after ingestion), while plasma levels of the aromatic amino acids (AAAs; Tyr, Phe, and Trp), basic amino acids (His, Lys, Arg), Asn, Glu, Pro, Cit, ABA, and Met declined to the same levels as those of the meal N-ingested group (Table 4).

However, the plasma levels of Tau, Gln, Gly, Ala, and Cys were not elevated by meal H ingestion (Table 4). Although not significant level, plasma levels of His and Gly declined less in the meal H ingested group than in the control group (Table 4). Two-way ANOVA also demonstrated that a significant effect of interaction term, i.e., meal×time was observed in all amino acids except for Tau, and a significant effect of meal was observed in Thr, Cit, Val, Met, Ile, Leu, Tyr, Orn, Lys, and Arg (data not shown).

In clinical examination, it is most important that the levels of PFAA concentration are stable throughout the blood sampling time, in addition to other indices of clinical tests. In the meal H-ingested group, a drastic alteration was observed in the meal H-ingested group (Fig. 1). Compared to meal N-ingested group, a significant effect of sampling time was observed in all amino acids (data not shown).

### High Protein Diet Ingestion Affects the Postprandial PFAA Profile Based Indices

**Figure 2:**
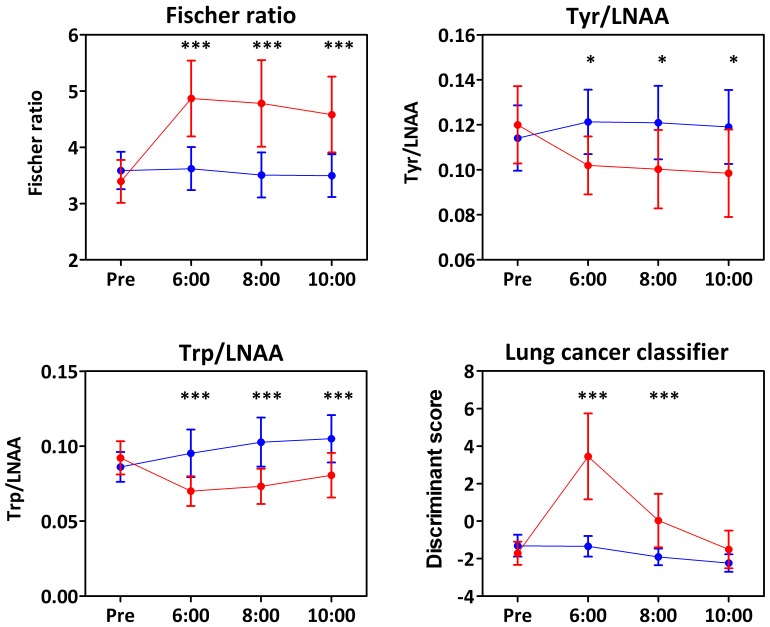
Preprandial and postprandial trends of the PFAA-based indices of meal N-ingested subjects (blue lines) and meal H-ingested subjects (red lines). *: Significant at p<0.05 with the Bonferroni multicomparison test after 2-way ANOVA. **: Significant at p<0.01 with the Bonferroni multicomparison test after 2-way ANOVA. ***: Significant at p<0.001 with the Bonferroni multicomparison test after 2-way ANOVA.

In other cases, in particular for most of the essential amino acids, imbalances between the increase of amino acid ingestion originating from dietary protein and plasma levels were still observed at 8, 10, and 12 hours after ingestion. For example, apparent imbalances in the neutral amino acids were observed. The plasma Fischer ratio in meal H-ingested subjects was higher than that of meal N-ingested subjects (Fig. 2A), and this is partially attributed to the fact that the BCAAs were not catabolized in the liver [26], [33]. Inversely, plasma Trp per sum of large neutral amino acids (LNAAs; Val, Ile, Leu, Tyr, and Phe) and Tyr/LNAAs were decreased in meal H-ingested subjects (Fig. 2B and Fig. 2C). Furthermore, scores of lung cancer classifier, which was the multiple logistic regression function composed of six amino acids (Ala, Val, Ile, His, Trp, and Orn) were significantly elevated (i.e. higher probability of lung cancer) in meal H-ingested subjects (Fig. 2D). Therefore, it was demonstrated that overnight postprandial PFAA profiles after ingestion of meal H-ingestion would result wrong decision in terms of health condition even in normal subjects.

### Intake Amount of Individual Amino Acids did not Reflect the Alteration in the PFAA Profile

**Figure 3:**
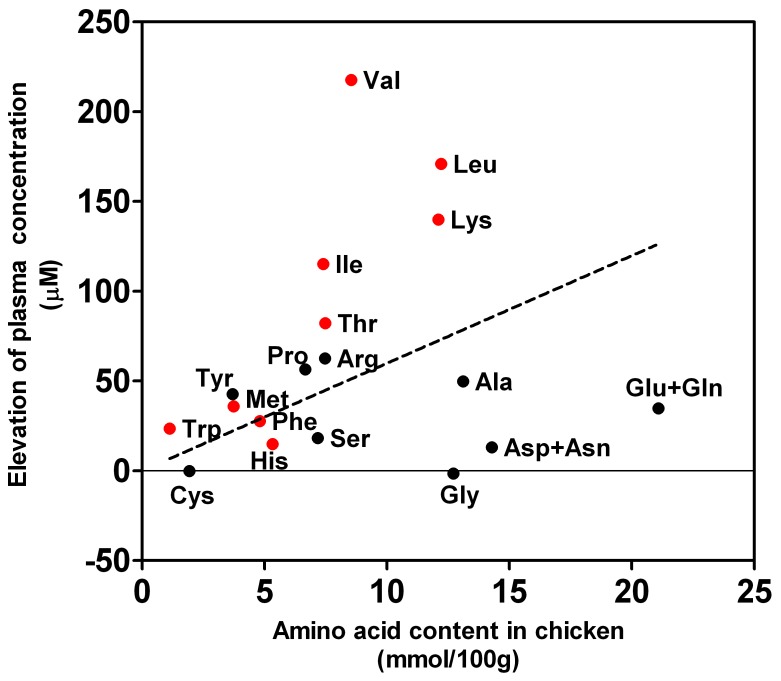
The relationship between the amino acid composition of chicken and the increase of the average plasma amino acid concentration observed in meal H-ingested subjects at 6∶00 am. Black symbols indicate non-essential amino acids (NEAAs), and red symbols indicate essential amino acids. The line indicates the linear regression line for all amino acids.

Next, the balance between the amount of ingested amino acids and the PFAA concentrations was investigated. As described in ***Subjects and Methods***, the most dominant part of the protein ingestion was attributed to the increase in the amount of chicken for high protein meal-ingested subjects. Therefore, the amino acid composition of chicken (http://www.mext.go.jp/b_menu/shingi/gijyutu/gijyutu3/houkoku/1298881.htm) may reflect the uptake of each amino acid. However, there was only a poor correlation between the increase in ingested amino acids and PFAA concentrations (Fig. 3). The non-essential amino acids Ala, Gly, Asp+Asn, and Glu+Gln showed an increase in plasma levels that was apparently repressed in comparison to the increase in ingestion. Inversely, in the case of BCAAs and Lys, a relatively higher increase in plasma levels was observed in comparison to the increase of ingestion (Fig. 3). The same tendency was observed at 8∶00 am and 10∶00 am (data not shown).

## Discussion

In this study, we found that a high-protein meal in the evening that contained 40% of the daily energy would continue to affect the PFAA profiles the next morning in Japanese adult men. In previous studies, only the short-term or cumulative effects of dietary protein intake on PFAA profiles were investigated. Our study clearly demonstrated the acute effect of excess dietary protein ingestion on PFAA after overnight fasting. It also must be noted that excess dietary protein ingestion caused transient instability in the PFAA profile based indices for health monitoring (Fig. 2). For these reasons, the performance of reliable PFAA profiling after overnight fasting requires that an extraordinary meal for dinner on the day before a health examination should be avoided.

According to the results of the preprandial amino acid profiles, the PFAA profile was quite stable; however, no subjects were controlled for their meal or other lifestyle before the ingestion of the meal as long as they lived a balanced life, because no significant difference in the plasma level was observed between the same subjects at a three-week interval (Table 3).

According to the comparison of the PFAA profiles between meal N-ingested subjects and meal H-ingested subjects, if the subject had a high protein diet at dinner, the PFAA profile after overnight fasting was still affected by the meal at 12 hours after the meal (Fig. 1). Therefore, the PFAA profile did not reflect the subject's health condition but rather the acute effects of high protein ingestion. It was also demonstrated that the patterns of the impacts of high protein meal ingestion varied a great deal depending on the type of amino acid. For example, the plasma levels of several non-essential amino acids (Ser, Gln, Gly, Ala, and Cys) were not elevated or were (Asn, Glu, and Pro) slightly elevated, while those of some of the essential amino acids (The, Val, Ile, Leu, and Lys) were elevated considerably in meal H-ingested subjects in this study (Fig. 3). In previous studies, it has also been demonstrated that plasma levels rise acutely due to high protein meal ingestion [25], [26], [27], [30], [31], [32]. Therefore, the influence of high protein meal ingestion on the plasma levels of non-essential amino acids would be almost negated by overnight fasting.

On the other hand, especially among the essential amino acids mentioned above, an amino acid imbalance was also observed throughout the period after ingestion (Fig. 1). This may be partially attributed to the antagonism among these three amino acids [34]. Plasma Leu would be preferentially incorporated into muscle, thus preventing the incorporation of Val and Ile [34]. The results of this study reflect this mechanism. In addition, imbalances in neutral amino acids were observed, such as the Fischer ratio (Fig. 2). A higher plasma Fischer ratio was observed in the meal H-ingested group than that in the meal N-ingested group (Fig. 2A). This may be partially attributed to the fact that BCAAs are not catabolized in the liver [26], [33]. As the scores of lung cancer classifier were also elevated significantly, several non lung cancer subjects would be misdiagnosed incidentally as lung cancer under this condition (Fig. 2D). In this case, because sign of coefficient for each amino acid is different each other, it is not obvious the effects of changes of PFAA profile influence the discrimination score when plasma levels of several amino acids are altered [6].

On the contrary, plasma Trp/LNAA and Tyr LNAA decreased in the meal H-ingested group (Fig. 2B and C). Plasma Trp and Tyr levels are strictly regulated because these amino acids are related to brain serotonin and catecholamine synthesis, which affect the central nervous system; LNAAs also are incorporated into the brain competitively via the same transporter [26], . Therefore, it has been suggested that decreases in the Trp/LNAA ratio and the Tyr/LNAA ratio are attributed to the neuronal pathway regulatory systems. Furthermore,

At these periods, it was also observed that the plasma levels of urea cycle-related substances, such as Orn, remained at higher levels in the high protein-ingested experiments (Fig. 1 and Table 4). This demonstrates that the transamination system via urea-cycle is still activated in the liver at 12 hours after the ingestion of a high protein meal to remove excess amine into the urine.

The results obtained in this study are still insufficient because PFAA levels were regulated not only as described in this section, but also by other regulatory networks [35], [36], [37], [38], and the network system might be affected by various factors, such as the ingested amino acid balance originating from dietary protein, the digestibility of the protein, and the amount of free amino acids [39], [40]. Further investigation is now ongoing to elucidate the overall mechanisms of PFAA regulatory systems.
